# Structural insights into DNA degradation by human mitochondrial nuclease MGME1

**DOI:** 10.1093/nar/gky855

**Published:** 2018-09-21

**Authors:** Chun Yang, Ruiqi Wu, Hehua Liu, Yiqing Chen, Yanqing Gao, Xi Chen, Yangyang Li, Jinbiao Ma, Jixi Li, Jianhua Gan

**Affiliations:** 1 State Key Laboratory of Genetic Engineering, Collaborative Innovation Center of Genetics and Development, Department of Physiology and Biophysics, School of Life Sciences, Fudan University, Shanghai 200433, China; 2 State Key Laboratory of Genetic Engineering, Collaborative Innovation Center of Genetics and Development, Department of Biochemistry, School of Life Sciences, Fudan University, Shanghai 200433, China; 3 Department of Neurology, Huashan Hospital, Fudan University, Shanghai 200040, China

## Abstract

Mitochondrial nucleases play important roles in accurate maintenance and correct metabolism of mtDNA, the own genetic materials of mitochondria that are passed exclusively from mother to child. MGME1 is a highly conserved DNase that was discovered recently. Mutations in MGME1-coding gene lead to severe mitochondrial syndromes characterized by external ophthalmoplegia, emaciation, and respiratory failure in humans. Unlike many other nucleases that are distributed in multiple cellular organelles, human MGME1 is a mitochondria-specific nuclease; therefore, it can serve as an ideal target for treating related syndromes. Here, we report one *Hs*MGME1-Mn^2+^ complex and three different *Hs*MGME1-DNA complex structures. In combination with *in vitro* cleavage assays, our structures reveal the detailed molecular basis for substrate DNA binding and/or unwinding by *Hs*MGME1. Besides the conserved two-cation-assisted catalytic mechanism, structural analysis of *Hs*MGME1 and comparison with homologous proteins also clarified substrate binding and cleavage directionalities of the DNA double-strand break repair complexes RecBCD and AddAB.

## INTRODUCTION

MGME1 (also known as DDK1) is a highly conserved, mitochondria-specific DNase (1). Like the mitochondrial nuclease DNA2, MGME1 belongs to the PD-(D/E)XK nuclease superfamily. However, in contrast to DNA2 (2–5) and many other mitochondrial DNases (6–13), MGME1 can cleave various types of DNA substrates with a preference for single-stranded DNAs (ssDNA) (14–16). *In vitro* studies have shown that MGME1 can cleave ssDNAs in both 5′→3′ and 3′→5′ directions. As revealed by loss of function mutations (1), MGME1-deficient cells showed apparent accumulation of 7S DNA, an ssDNA that synthesized by DNA POLγ. 7S DNA can forms displacement loop (D-loop) structure with the non-coding region (NCR) of mtDNAs (17,18). MGME1 has no ribonuclease activity; when a chimeric DNA-RNA substrate is provided, MGME1 will slide over the RNA fragment and cleave the DNA only. In addition to ssDNA and DNA-RNA chimeric oligonucleotides, MGME1 can also cleave double-stranded DNAs (dsDNAs) and DNAs with a 5′-flap or 3′-flap (16).

*Hs*MGME1 does not interact with the commonly known factors involved in mtDNA transactions (such as the DNA-interacting proteins TFAM and mtSSB of the mitochondrial nucleoid), but recent studies (14,19,20) suggested that *Hs*MGME1 could form a complex with PolgA, a catalytic subunit of the mtDNA polymerase. The interaction with PolgA suggests that MGME1 is a potential constituent of the mitochondrial replisome. Recent studies also suggested that *Hs*MGME1, PolgA and TWINKLE (a mitochondrial DNA helicase) can work together in the double-strand degradation process of mtDNA (21,22). Like many known mitochondrial nucleases, the cleavage activity of MGME1 is important for maintenance of the mitochondrial genome. However, while other mitochondrial nucleases are mainly involved in the repair process of mtDNA, MGME1 participates in both replication and double-strand degradation processes of mtDNA. MGME1 is encoded by the gene *C20orf72* in humans. Loss-of-function mutations in the MGME1-coding gene can lead to severe multisystem mitochondrial disorders in humans with depletion and rearrangements of mtDNA (16). Patients with loss-of-function MGME1 mutations could develop progressive external ophthalmoplegia, skeletal muscle wasting/weakness, respiratory distress, emaciation, severe dilated cardiomyopathy, mental retardation, microcephalus, and severe gastrointestinal symptoms (21,22).

Based on the structures of other PD-(D/E)XK nucleases, several MGME1 models have been proposed previously (1,16); however, due to the lack of high-resolution structures, especially the structures of MGME1 in complex with DNA, detailed information underlying substrate binding and cleavage of MGME1 remains elusive. Herein, we report crystallographic and *in vitro* catalytic studies of MGME1. In total, four MGME1 structures were solved: one is in a complex with Mn^2+^ and the other three are complexed with DNA substrates, with or without Ca^2+^. Structural analysis and comparison suggest that MGME1 follows a two-cation-assisted catalytic mechanism of substrate cleavage. In addition to DNA binding, our structures also revealed one unique structural pin; the pin is composed of two aromatic residues (Trp152 and Phe173) and is important for DNA duplex unwinding and flap DNA cleavage of MGME1. MGME1 shares both sequence and structural similarities with the RecB-type nucleases; the comparison also elucidated substrate binding and the catalytic mechanism of RecB and AddA proteins, which are important components of the DNA double-strand break repair complexes RecBCD (23–25) and AddAB (26,27), respectively.

## MATERIALS AND METHODS

### Plasmid construction

Full-length wild-type (WT) *Hs*MGME1 was obtained by PCR using a human cDNA library as a template. The PCR products were cleaved with enzymes BamHI and XhoI and cloned into a pET28-Sumo vector. The recombinant vector was then transferred into *Escherichia coli* BL21 (DE3) competent cells for protein expression. All the truncated or mutated *Hs*MGME1 were constructed by PCR or overlap PCR using WT *Hs*MGME1 as a template. Primers used in plasmid construction are listed in Supplementary Table S1. Sequences of plasmids were confirmed by DNA sequencing. All recombinant strains were protected using 30% glycerol and stored in a −80°C freezer prior to use.

### Protein expression and purification

All *Hs*MGME1 proteins were expressed overnight in BL21 (DE3) cells with lysogeny broth (LB) medium supplemented with 50 μg/ml kanamycin at 37°C. When OD_600_ reached 0.6, expression was induced by adding isopropyl β-D-1-thiogalacto-pyranoside (IPTG) at a final concentration of 0.2 mM for 18 h at 18°C. The cells were harvested by centrifugation and the pellets were stored at −80°C.

For overproduction of the Se-Met substituted WT *Hs*MGME1, the revived recombinant strains from 20 ml overnight cultures were inoculated into 2 l of LB medium supplemented with kanamycin (50 μg/ml) and grown at 37°C. Cells were harvested by centrifugation when OD_600_ was 0.4, then the pellets were washed and resuspended in M9 medium (47.7 mM Na_2_HPO_4_, 22 mM KH_2_PO_4_, 8.6 mM NaCl, and 28.2 mM NH_4_Cl). The resulting cells were transferred into 2 l fresh M9 medium supplemented with 50 μg/ml kanamycin and 40 mg/l Se-Met (J&K). After growing at 37°C for 1 h, protein expression was induced by adding IPTG at a final concentration of 0.2 mM. The induced cultures were then grown at 18°C for an additional 18 h and the cells were harvested by centrifugation.

To improve solubility, the N-terminal MTS sequence (residues 1–20) was deleted in all WT and mutant proteins of *Hs*MGME1. All proteins were purified using the same protocol. The pellets were first resuspended in Ni binding buffer (Buffer A, 20 mM Tris pH 8.0, 500 mM NaCl and 25 mM imidazole pH 8.0) and lysed under high pressure with a JN-02C cell crusher. The homogenate was clarified by centrifugation and the supernatant was loaded onto a HisTrap™ HP column (GE Healthcare) equilibrated with Buffer A. The His-Sumo-*Hs*MGME1 fusion proteins were eluted from the column using elution buffer (20 mM Tris pH 8.0, 500 mM NaCl and 500 mM imidazole pH 8.0) with a gradient. Fractions containing the desired fusion proteins were pooled and dialyzed against Buffer S (20 mM Tris pH 8.0, 500 mM NaCl, and 25 mM imidazole pH 8.0) at 4°C for 3 h; Ulp1 protease was also added to the sample during the dialysis process. The sample was again loaded onto the HisTrap™ HP column and the flow-through containing the target proteins was collected and mixed with Tris buffer (20 mM Tris pH 8.0) to dilute the NaCl to a final concentration of 200 mM. The diluted sample was applied to a HiTrap Q HP column equilibrated with Q binding buffer (20 mM Tris pH 8.0, 200 mM NaCl). The flow-through containing the target proteins were concentrated and loaded onto a Hi 16/60 Superdex G200 column (GE Healthcare) equilibrated with a gel filtration buffer (20 mM Tris pH 8.0, 200 mM NaCl and 1 mM DTT). Protein purity was analyzed using an SDS-PAGE gel and the samples were concentrated and snap-frozen using liquid nitrogen and stored at −80°C until use.

### Crystallization and data collection

The initial crystallization conditions of all *Hs*MGME1 crystals were identified using a Gryphon crystallization robot system (Arts Robin Instrument) with commercial kits at 18°C. The sitting drop vapor diffusion method was used during the initial screening, whereas all crystallization optimization processes were done using the hanging drop vapor diffusion method. Protein concentrations were 10 mg/ml for all samples, and the CaCl_2_ concentration was 30 mM for the H180Q-DNA2-Ca^2+^ complex. The protein: DNA molecular ratio was 1:1.2 for all *Hs*MGME1-DNA complexes. Prior to crystallization, all samples were incubated on ice for 30 min. The crystallization conditions were 0.35 M di-sodium tartrate, 21% PEG 3350 and 0.1 M MnCl_2_, 0.1 M phosphoric-citric acid pH 4.2 and 20% PEG300 and 0.1 M CHES pH 9.5 and 20% PEG 8000 for *Hs*MGME1-Mn^2+^, *Hs*MGME1-DNA2, and H180Q-DNA2-Ca^2+^ complexes, respectively. Both native and Se-Met substituted *Hs*MGME1-DNA3 complex crystals were grown in 0.1 M HEPES pH 7.5, 0.8 M NaH_2_PO_4_, and 0.8 M K_2_HPO_4_. The drop contained equal volumes of sample and crystallization solution for all crystals.

All crystals were cryoprotected using their mother liquor supplemented with 25% glycerol and snap-frozen in liquid nitrogen. X-ray diffraction data were collected on beamlines BL17U1 and BL19U1 at the Shanghai Synchrotron Radiation Facility (SSRF) at cryogenic temperatures and maintained with a cryogenic system. Data processing was carried out using HKL2000 or HKL3000 programs (28). The data collection and processing statistics are summarized in Supplementary Table S2.

### Structure determination and refinement

The Se-*Hs*MGME1-DNA3 structure was solved using the single-wavelength anomalous diffraction (SAD) method (29) with the AutoSol program (30) embedded in the Phenix suite (31). The initial model that covered approximately 70% of protein residues in the asymmetric unit was built with the Autobuild program. The model was then refined against diffraction data of the native *Hs*MGME1-DNA3 crystal with the Refmac5 program (32) of CCP4i, which revealed the detailed orientations of the missing residues. During refinement, 5% of randomly selected data was set aside for free R-factor cross validation calculations. The 2*F*_o_ – *F*_c_ and *F*_o_ – *F*_c_ electron density maps were regularly calculated and used as guides for building the missing amino acids, DNA and solvent molecules with COOT (33). The *Hs*MGME1-Mn^2+^, *Hs*MGME1-DNA2 and H180Q-DNA2-Ca^2+^ complex structures were all solved using the molecular replacement method with the Phaser program of CCP4i suite using the *Hs*MGME1-DNA3 structure as the search model. The final refinement of all structures was done with the phenix.refine program (34) of Phenix. The structural refinement statistics are summarized in Supplementary Table S2.

### *In vitro* cleavage assays

6-Carboxy-fluorescein (FAM)-labelled substrates (Supplementary Table S3) were purchased from Generay Biotech Ltd. (Shanghai, China). To obtain double-stranded substrates, self-complementary oligonucleotides were dissolved in reaction buffer (20 mM Tris pH 8.0, 100 mM NaCl) and heated at 95°C for 15 min followed by slow cooling to room temperature.

The standard enzymatic assay was performed in a 20 μl mixture containing 0.8 μM of DNA substrates, 0.1 mg/ml bovine serum albumin (BSA), 100 mM NaCl, 5 mM MgCl_2_, 20 mM Tris–HCl (pH 8.0), 1 mM DTT, and different concentrations of WT or mutant *Hs*MGME1. Reaction mixtures were incubated at 37°C for the indicated time and terminated by adding an equal volume of formamide loading dye (95% formamide, 20 mM ethylenediaminetetraacetic acid, 0.025% SDS, 0.02% bromophenol blue, and 0.02% xylene cyanol in 1× TBE buffer). The mixtures were incubated at 95°C for 10 min and analyzed on denaturing 18% polyacrylamide 8 M urea gels. The gel was imaged using Typhoon FLA 9000. The intensities of the bands were quantified by ImageQuantTL and the data was compared in GraphPad Prism.

### Crosslinking assays

Four-microliter samples consisting of 1 μl 5 × reaction buffer (150 mM HEPES–NaOH pH 7.6, 150 mM NaCl, 5 mM DTT and 0.5 mM EDTA), 2 μl *Hs*MGME1 protein (50 μM), and 1 μl DNA3 (10 μM) were mixed and incubated at room temperature for 30 min. One-microliter diluted DSS (disuccinimidyl suberate) was added to the reaction system at final concentrations of 50, 100 and 200 μM. The samples were incubated at room temperature for an additional 20 min and quenched by the addition of 1.25 μl SDS-loading buffer. Samples were heated at 95°C for 5 min, centrifuged, and loaded onto 10% SDS-PAGE gels. Gels were run at 200 V for 60 min in 1× SDS running buffer and stained with Coomassie brilliant blue.

## RESULTS

### Overall structure of *Hs*MGME1

**Figure 1.**
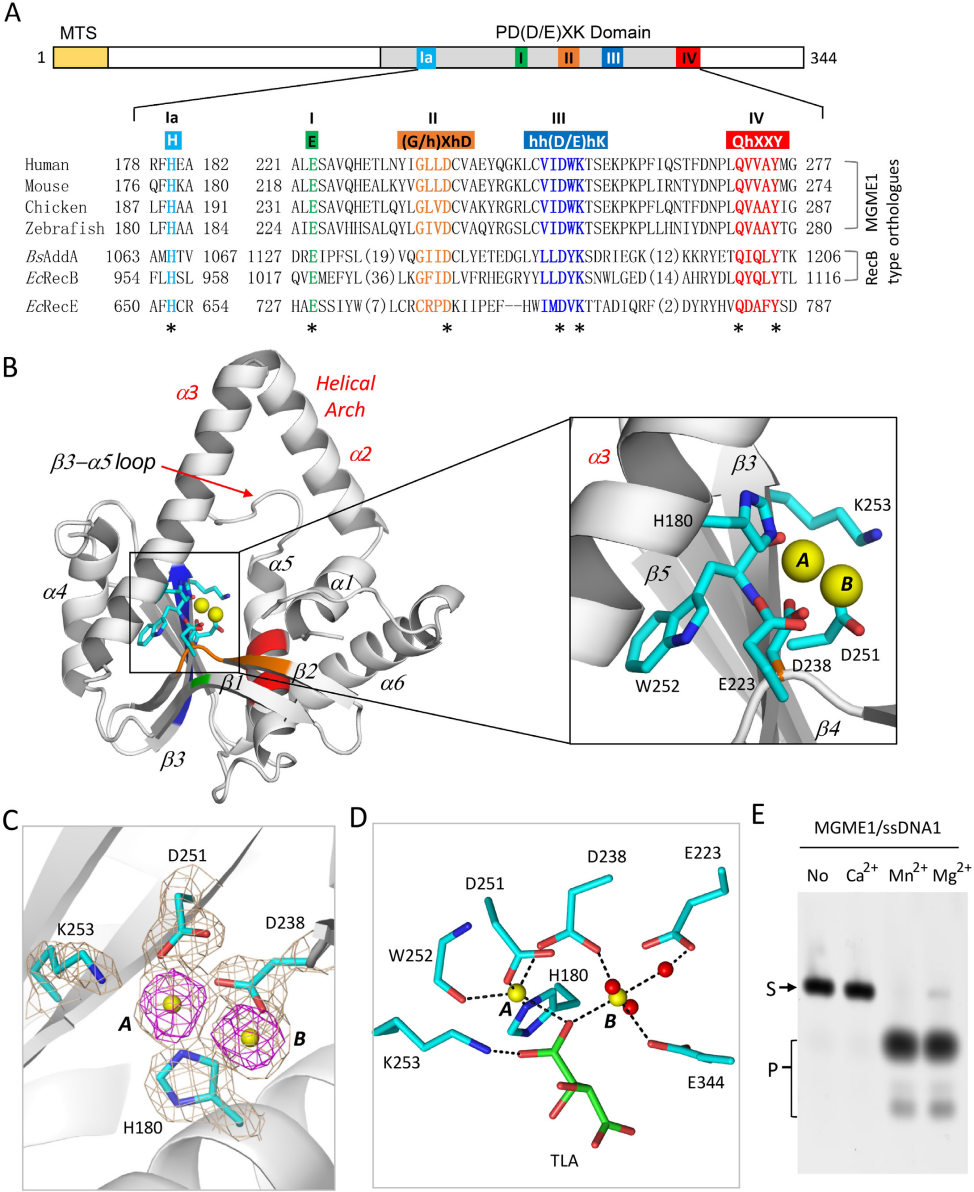
*Hs*MGME1 is a cation-dependent nuclease. (**A**) Domain architecture of *Hs*MGME1. MTS indicates the mitochondrial targeting signal. Residues of the conserved motifs are highlighted in bold. (**B**) Overall structure of the *Hs*MGME1-Mn^2+^ complex. *Hs*MGME1 is shown as cartoon in white. (**C**) 2*F*_o_ – *F*_c_ electron density of Mn^2+^ and surrounding residues. The wheaten and magenta maps are contoured at 1.5 and 5.0 σ level, respectively. (**D**) Detailed coordination of Mn^2+^. TLA stands for tartrate acid present in the crystallization condition. (**E**) *in vitro* cleavage assay showing the effect of cations. *Hs*MGME1, ssDNA1, and cations (if present) concentrations are 0.1 μM, 0.8 μM and 5 mM, respectively. The conserved motifs Ia, I, II, III and IV are highlighted in identical colors in panels A and B. In (B–D), Mn^2+^ and coordinating water molecules are shown as yellow and red spheres, respectively. Active site residues are shown as sticks in atomic colors (C, cyan; N, blue; O, red).

Human MGME1 (*Hs*MGME1) protein is composed of 344 amino acids and belongs to the PD-(D/E)XK nuclease superfamily. In addition to the C-terminal nuclease domain, *Hs*MGME1 has one predicted mitochondria-targeting sequence (MTS) at the N-terminus (Figure 1A). Interestingly, besides the three common PD-(D/E)XK motifs (I, II and III), *Hs*MGME1 shares two additional characteristic motifs (Ia and IV) with RecB-type nucleases, such as *Bs*AddA and *Ec*RecB. Motif Ia contains one highly conserved histidine residue, corresponding to His180 in *Hs*MGME1; motif IV is composed of five residues QhXXY, where h represents a hydrophobic residue and X represents any residue. *Hs*MGME1 shares very high (up to 83%) sequence similarity with MGME1 orthologs from other species, such as mouse, chicken, and zebrafish, but has very low similarity with other PD-(D/E)XK nucleases.

MGME1 plays an important role in mitochondrial genome maintenance, however, no MGME1 structure was available. To better understand the structure and function of MGME1, we carried out crystallographic studies using *Hs*MGME1. In total, four *Hs*MGME1 structures were solved, including one apo- structure and three DNA-bound complex structures (Supplementary Table S2). The apo-*Hs*MGME1 structure belongs to the *P*4_3_2_1_2 space group and contains one *Hs*MGME1 molecule per asymmetric unit (Figure 1B); the monomeric state of *Hs*MGME1 is consistent with our gel filtration profile and a previous report (1). As revealed by all four structures, *Hs*MGME1 contains five β-strands (β1-β5) and six α-helices (α1–α6). The two long helices α2 and α3 are formed by the residues 143–160 and 162–187, respectively. α2 and α3 are assembled in a helical arch; the angle between the two helices is ∼70°. In the apo-*Hs*MGME1 structure, residues 1–97, 113–117 and 190–198 are disordered (Supplementary Figure S1A); the β3–α5 connecting loop (aa 255–264) resides right under the helical arch (Figure 1B and Supplementary Figure S1B). The side chain of Phe261 forms a weak hydrophobic stacking interaction with the side chains of Trp152 and Lys153 of α2 (Supplementary Figure S1B); the backbone O atoms of Lys257 and Pro258 form water-mediated hydrogen bond (H-bond) interactions with Tyr168 and Thr169 of α3 (Supplementary Figure S1B), respectively. These interactions may help stabilize the conformation of both the helical arch and the β3-α5 connecting loop.

### Mn^2+^ coordination of *Hs*MGME1

A previous study (1) showed that the catalytic activity of *Hs*MGME1 was dependent on a divalent cation, commonly Mg^2+^. Though Mg^2+^ was not present in either the crystallization sample or the crystallization buffer, the crystallization buffer of the apo-*Hs*MGME1 crystals contained 100 mM Mn^2+^. As depicted in Figure 1C and D, two well-defined Mn^2+^ ions were captured at the active site of the apo-*Hs*MGME1 structure, which was hereafter referred to as *Hs*MGME1-Mn^2+^. The A-site Mn^2+^ was five-coordinated. In addition to the O1 atom of the tartrate acid (TLA, which was present in the crystallization buffer) and the main chain O atom of Trp252, it was also coordinated by the side chain NE2, OD2 and OD1 atoms of His180, Asp238 and Asp251, corresponding to the highly conserved His, Asp, and Asp residues of motifs Ia, II and III, respectively. The B-site Mn^2+^ was six-coordinated. In addition to three water molecules and the side chain OE2 atom of Glu344 (the last residue of *Hs*MGME1), the B-site Mn^2+^ was also coordinated by TLA and Asp238, via their O1 and OD1 atoms, respectively. The average coordinating distances of the A-site and B-site Mn^2+^ ions were all ∼2.15 Å.

As confirmed by both structures and catalytic assays, many Mg^2+^-dependent nucleases, including RNase III (35) and RNase H (36,37), are also active in the presence of Mn^2+^. To test whether Mn^2+^ can also support the cleavage activity of *Hs*MGME1, we performed an *in vitro* catalytic assay using wild type (WT) *Hs*MGME1 and one short 12-nt ssDNA (ssDNA1, 5′-TTCTTCTTCTTC-3′, which was FAM-labelled at the 5′-end). As depicted in Figure 1E, *Hs*MGME1 is inactive in the absence of divalent cations, but it can efficiently cleave DNA1 in the presence of either Mg^2+^ or Mn^2+^, indicating that Mn^2+^ can mimic Mg^2+^ in substrate binding and cleavage of *Hs*MGME1. During the *in vitro* ssDNA cleavage assays (Supplementary Figure S2), we noticed that *Hs*MGME1 had some weak cleavage site preference; altering the compositions of the ssDNAs will lead to corresponding changes in product patterns.

### ssDNA recognition of *Hs*MGME1

**Figure 2.**
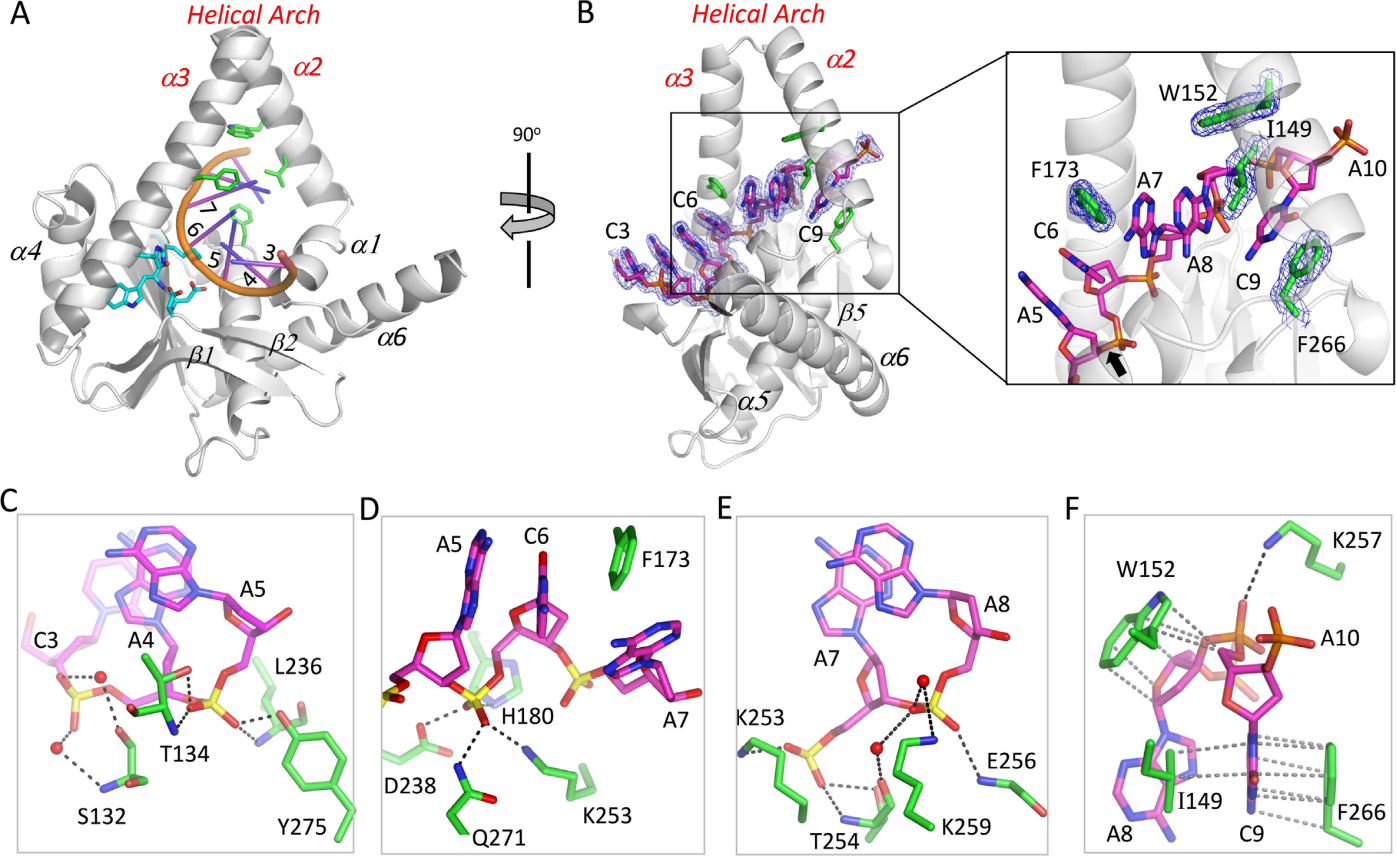
Single-stranded DNA recognition by *Hs*MGME1. (**A**) and (**B**) Overall fold of the *Hs*MGME1-ssDNA2 complex. Active site residues are shown as sticks in atomic colors (C, cyan; N, blue; O, red) in (A). In (B), 2*F*_o_ – *F*_c_ electron density maps are contoured at 1.5 σ level. Bending of the DNA backbones are shown in the right and the O-P bond that will be cleaved by *Hs*MGME1 is indicated by black arrow. (**C**–**F**) Detailed interactions between *Hs*MGME1 and the individual nucleotide of ssDNA2. H-bonds and hydrophobic interactions are indicated by black and gray dashed lines, respectively. Water molecules are shown as red spheres. ssDNA2 and ssDNA2-interacting residues are shown as sticks with their C-atoms colored in magenta and green in (C–F).

Among all substrates, *Hs*MGME1 has a strong preference for ssDNAs. Details of ssDNA recognition were revealed by the *Hs*MGME1–ssDNA2 complex (Figure 2A), which was determined at high resolution (1.75 Å). ssDNA2 (5′-AACAACAACAAC-3′) is 12-nt in length and resides under the helical arch in the structure. As revealed by the structural superposition (Supplementary Figure S1C), binding of ssDNA2 has no obvious impact on the conformation of the helical arch, but it causes significant conformational changes to the β3–α5 connecting loop.

Due to structural disorder, the 5′-end A1A2 and 3′-end A11C12 were not observed, whereas the central seven nucleotides (from C3 to A9) and the phosphate group of A10 of ssDNA2 are all well defined in the *Hs*MGME1–ssDNA2 structure (Figure 2B). C3, A4, A5 and C6 of ssDNA2 are arranged in an A-form like conformation. C3 mainly forms a stacking interaction with A4. In addition to the stacking interactions between their nucleobases, A4, A5 and C6 also form extensive H-bond interactions with *Hs*MGME1. The phosphate group of A4 (Figure 2C) forms two water-mediated H-bonds with the main chain N and side chain OG atoms of Ser132. A5 forms four direct H-bonds (Figure 2C). The O1P atom of A5 forms one H-bond (2.9 Å) with the main chain N atom of Leu236 and one H-bond (2.6 Å) with the side chain OH atom of Tyr275. Via its main chain N atom and side chain OG1 atom, Thr134 forms two H-bonds with the O2P atom of A5; the average H-bond distance is 2.8 Å. Like A5, the phosphate group of C6 (Figure 2D) also forms four direct H-bonds with *Hs*MGME1, via the NE2 atom of His180, the OD1 atom of Asp238, the NZ atom of Lys253, and the NE2 atom of Gln271.

The backbone of ssDNA2 is severely bent in-between C6 and A7, resulting in an almost perpendicular orientation between the nucleobases of C6 and A7 (Figure 2B, right panel). The nucleobases of A7 and A8 stack with each other. Via their phosphate groups, A7 and A8 form extensive H-bond interactions with *Hs*MGME1 (Figure 2E). The O1P atom of A7 forms two H-bonds (2.8 and 2.7 Å) with Thr254, via the main chain N atom and side chain OG1 atom, respectively; the O2P atom of A7 forms one H-bond (2.8 Å) with the side chain NZ atom of Lys253. In addition to the direct H-bond (2.8 Å) between the O1P atom of A8 and the main chain N atom of Glu256, A8 also forms two water-mediated H-bonds with the main chain O atom of Thr254 and the side chain NZ atom of Lys259, respectively.

Interestingly, the backbone of ssDNA2 is also bent in-between A8 and C9 and the nucleobases of A8 and C9 are almost perpendicular to each other (Figure 2B and F). The side chain of Trp152 forms several hydrophobic interactions with the sugar pucker of A8 and the backbone C5* atom of C9. The nucleobase of C9 is sandwiched in between the side chains of Ile149 and Phe266, forming hydrophobic stacking interactions with them, especially Phe266. Both Ile149 and Trp152 reside at the middle of helix α2. The conformation of C9 was further stabilized by one H-bond (2.8 Å) between its O1P atom and the NZ atom of Lys257.

### Active site comparison of *Hs*MGME1

As confirmed by our *in vitro* catalytic assay (Figure 1E), *Hs*MGME1 has no cleavage activity in the presence of Ca^2+^, a common inhibitor of Mg^2+^-dependent nucleases. Ca^2+^ has been extensively utilized in the structural and mechanistic studies of various nucleases (38). Surprisingly, though 10 mM CaCl_2_ was included in the crystallization sample, no Ca^2+^ was bound at the active site of the *Hs*MGME1-ssDNA2 complex. Unlike many nucleases, which coordinate Mg^2+^ (or Mn^2+^) through the side chains of three or more negatively charged residues (Asp or Glu), *Hs*MGME1 coordinates Mn^2+^ through the side chains of His180, Asp238, and Asp251, and the main chain of Trp252 (Figure 1D). The NE2 atom of His180 forms one strong H-bond (2.7 Å) with the backbone O atom of Trp252 in the *Hs*MGME1–ssDNA2 structure; compared to the *Hs*MGME1–Mn^2+^ structure, the Trp252–Lys253 amide plane was tilted about 20° in the *Hs*MGME1-ssDNA2 structure (Supplementary Figure S1D).

**Figure 3.**
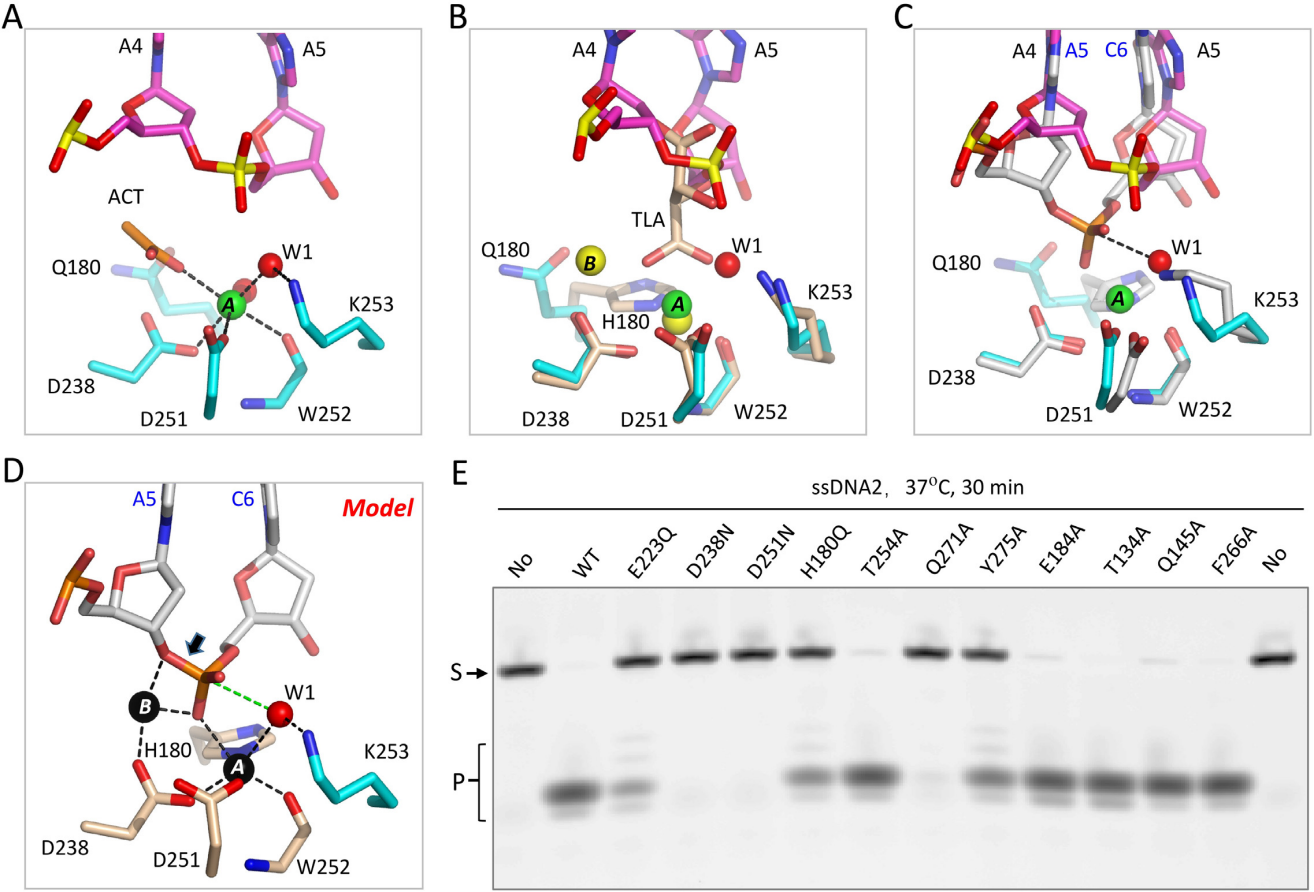
Catalytic site assembly of *Hs*MGME1. (**A**) Catalytic site conformation of H180Q–ssDNA2–Ca^2+^. ACT stands for acetic acid present in the crystallization condition. Ca^2+^ and Ca^2+^-coordinating water molecules are shown as green and red spheres. DNA and catalytic site residues are shown as sticks with C-atoms colored in magenta and cyan, respectively. (**B**) Superposition of the catalytic site structures of H180Q-ssDNA2-Ca^2+^ and *Hs*MGME1-Mn^2+^. In *Hs*MGME1-Mn^2+^, Mn^2+^ ions are shown as yellow spheres, TLA and Mn^2+^-coordinating residues are shown as sticks with C-atoms colored in wheaten. (**C**) Superposition of the catalytic site structures of H180Q–ssDNA2-Ca^2+^ and *Hs*MGME1–ssDNA2. C-atoms are colored in white in the *Hs*MGME1-ssDNA2 complex. (**D**) Proposed model of the catalytic form *Hs*MGME1, DNA, and cation ternary complex. The O-P bond that will be cleaved by *Hs*MGME1 is indicated by black arrow. (**E**) *in vitro* cleavage assay using ssDNA2 and WT or mutated *Hs*MGME1. Substrates and products are labelled as S and P, respectively.

We speculated that the direct His180–Trp252 interaction disrupts Ca^2+^ binding and leads to the associated conformational changes of Asp251 and Lys253 in the structures (Supplementary Figure S1D). To test our hypothesis, we constructed one *Hs*MGME1 mutant with His180 substituted by Gln180, which is similar to His180 in size but neutral in charge. Using this H180Q mutant, we successfully solved one *Hs*MGME1 ternary complex structure, H180Q–ssDNA2–Ca^2+^. As depicted in Figure 3A, one six-coordinated Ca^2+^ ion was bound at the active site of *Hs*MGME1. The Ca^2+^ ion coordinates with the O atom of acetic acid (ACT), the side chain OD2 atom of Asp238, the side chain OD1 atom of Asp251, the main chain O atom of Trp252, as well as two water molecules. Beside Ca^2+^-coordination, water molecule W1 also forms one H-bond (2.6 Å) with the side chain NZ atom of Lys253.

The residue Gln180 does not coordinate Ca^2+^; compared with His180 in the *Hs*MGME1–Mn^2+^ structure, the side chain of Gln180 is rotated clockwise around the CA-CB bond for about 150° in the H180Q-ssDNA2-Ca^2+^ complex (Figure 3B). Though they are not identical in coordination, the orientations of the Ca^2+^ and the A-site Mn^2+^ are very similar in the H180Q-ssDNA2-Ca^2+^ and *Hs*MGME1–Mn^2+^ structures. Interestingly, the orientations of the Ca^2+^-coordinating water molecule W1 and the O11 atom of TLA are also very similar in the two structures; however, compared to *Hs*MGME1–Mn^2+^, the NZ atom of Lys253 was shifted about 0.6 Å in the H180Q–ssDNA2–Ca^2+^ complex.

Binding of Ca^2+^ does not affect the folding of *Hs*MGME1. As indicated by the very low root mean square deviation value (rmsd, 0.7 Å), the overall structure of H180Q–ssDNA2–Ca^2+^ is very similar to that of the *Hs*MGME1–ssDNA2 complex (Supplementary Figure S1E). However, detailed conformations of the two structures are not identical at their active sites (Figure 3C). Instead of forming an H-bond interaction, the NZ atom of Lys253 in the *Hs*MGME1-ssDNA2 structure almost overlaps with the Ca^2+^-coordinating water molecule W1 of the H180Q–ssDNA2–Ca^2+^ complex. Though one Ca^2+^ ion was bound at the A-binding site, the B-binding site was not occupied by any cations in the H180Q–ssDNA2–Ca^2+^ complex, which may have weakened the overall DNA coordinating ability of Ca^2+^ and led to the shifting of the A5 nucleotide.

### Catalytic mechanism and *in vitro* DNA degradation by *Hs*MGME1

The cleavage activity of *Hs*MGME1 is cation-dependent; though we did extensive screening, no structure with cations coordinated with both protein and DNA was obtained. To elucidate the catalytic mechanism of *Hs*MGME1, we further analyzed our structures and produced one plausible model (Figure 3D), in which the Mn^2+^ ions and Mn^2+^-coordinating residues (His180, Asp238, Asp251 and Trp252), water molecule W1 and Lys253, and DNA positions were directly taken from the *Hs*MGME1-Mn^2+^, H180Q-ssDNA2-Ca^2+^ and *Hs*MGME1-ssDNA2 complexes, respectively. In the model, both A-site and B-site Mn^2+^ ions coordinate with the O1P atom of the cleavage site nucleotide. The A-site Mn^2+^ ion also coordinates with the water molecule W1, which is in-line with the phosphorus atom of cleavage site nucleotide and O3* atom of the leaving nucleotide; the distance between W1 and the phosphorus atom of the cleavage site nucleotide is 3.0 Å. In addition to O1P of C6, the B-site Mn^2+^ ion also coordinates with the O3* atom of the leaving nucleotide.

The complex structures of many divalent cation (Mg^2+^ or Mn^2+^)-dependent nucleases, such as C3PO (38), RNase III (39), and RNase H (36), have been previously reported. Though the overall structures are very different, the *Hs*MGME1 model and these nucleases share remarkable similarity in cation-coordination, suggesting that *Hs*MGME1 also follows a two-cation-assisted mechanism in substrate degradation. Via deprotonation, the A-site cation will activate the water molecule W1, which will attack the phosphorus atom of the cleavage site nucleotide. In addition to assembly of the catalytic form complex, the B-site cation can also facilitate O–P bond breakage by neutralizing the negative charge developing on the leaving oxygen anion. Lys253 forms one H-bond and orients the nucleophilic water molecule W1 for in-line attack. As revealed by previous studies (38,39), mutation of Lys253 will significantly lower the catalytic activity of *Hs*MGME1. Like *Hs*MGME1 and all other PD-(D/E)XK superfamily members, the active sites of *Saccharomyces cerevisiae* Rnt1P (40), *Kluyveromyces polysporus* DCL1 (41), and many other nucleases also contain one Lys reside, which may use a similar mechanism in regulating the activities of the enzymes.

To verify the catalytic mechanism of *Hs*MGME1, we constructed several mutants with the cation-coordinating residues mutated, including H180Q, D238N, and D251N, and performed *in vitro* cleavage assays using 5′ FAM-labeled ssDNA2. As depicted in Figure 3E, ssDNA2 cleavage activity of the H180Q mutant was significantly reduced compared with the WT *Hs*MGME1. ssDNA2. More dramatic reduction was observed for the D238N and D251N mutants, suggesting that Asp238 and Asp251 are critical for the catalytic activity of *Hs*MGME1. Though it does not directly coordinate Mn^2+^, the invariant Glu223 residue of motif I forms one H-bond (2.7 Å) with one water coordinating with the B-site Mn^2+^ (Figure 1D). Replacing Glu223 with Gln223 (for E223Q mutant) significantly lowered the catalytic ability of the protein; the ssDNA2 cleavage efficiency of E223Q mutant is comparable to that of H180Q mutant. Together, these observations suggest that His180 and Glu223 mainly affect the coordination of the cations, whereas Asp238 and Asp251 are involved in both cation-coordination and catalytic process.

In addition to the cation-coordinating residues, we also mutated many residues involved in DNA-binding. As depicted in Figure 3E, replacing Thr134 or Phe266 with an Ala residue has no obvious impact on the protein's ssDNA2 cleavage activity. However, compared to the WT protein, the ssDNA2 cleavage activities of Q145A, E184A, and T254A mutants are all slightly weakened. More dramatic reduction was observed for the Q271A and Y275A mutants. Tyr275 interacts with the O1P atom of the nucleotide before the cleavage site. Though it interacts with the O2P atom in the *Hs*MGME1–ssDNA2 complex, Gln271 interacts with the O1P atom of the cleavage site nucleotide in the H180Q–ssDNA2–Ca^2+^ complex, which may facilitate catalytic complex assembly and contribute to its strong effect on the cleavage activity of *Hs*MGME1.

### Unique structural pin for duplex unwinding

**Figure 4.**
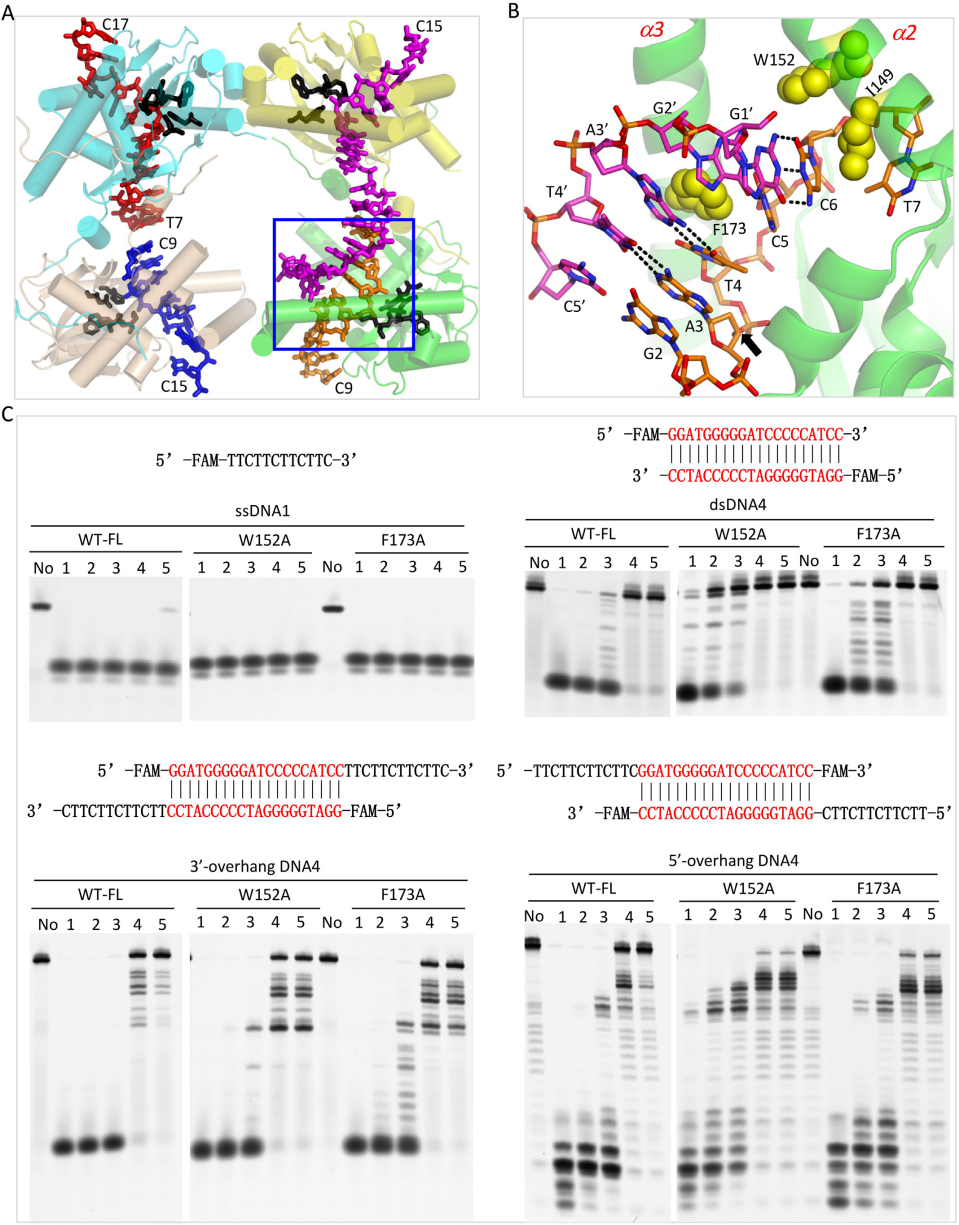
Functional characterization of the pin motif of *Hs*MGME1. (**A**) Overall structure of the *Hs*MGME1-DNA3 complex. *Hs*MGME1 and DNA molecules are shown as cartoon and sticks, respectively. The active site residues of all the four *Hs*MGME1 molecules are shown as black sticks. DNA duplex region is indicated by blue box. (**B**) Close-up view showing the detailed conformation of the duplex and pin residues, which are shown as sticks and spheres, respectively. The O-P bond that will be cleaved by *Hs*MGME1 is indicated by black arrow. (**C**) *in vitro* DNA cleavage assay using WT and pin region-mutated *Hs*MGME1. ssDNA1 concentration is 0.8 μM. WT or mutant *Hs*MGME1 concentrations are 0.8, 0.4, 0.2, 0.1 and 0.05 μM in lanes 1–5, respectively. No protein is present in the lane labelled with No.

*Hs*MGME1 can cleave various types of DNAs. In addition to ssDNA2, we also successfully solved one structure of *Hs*MGME1 in complex with DNA3 (5′-GGATCCTTCTTCTTCTTC-3′).) Via the GGATCC located at the 5′-end, DNA3 can form a 6-bp duplex with a 12-nt overhang on the 3′-ends of both strands. The *Hs*MGME1-DNA3 crystal belongs to the C2 space group and contains four *Hs*MGME1 molecules per asymmetric unit (Figure 4A). Three of the *Hs*MGME1 molecules only recognize the 3′-overhang region, whereas the last one interacts with both duplex and overhang regions of DNA3. As indicated by the low rmsd values (∼0.5 Å), the overall folds of the four *Hs*MGME1 molecules are very similar. Structural superposition revealed that folding of these proteins are also very similar to the one in the *Hs*MGME1-DNA2 complex (Supplementary Figure S3A).

DNA duplexes normally adopt a B-form conformation, however, unlike the regular DNA duplexes, the DNA3 duplex is severely distorted. As depicted in Figure 4B, the nucleobase of G2′ is almost perpendicular to that of A3′. Though the flanking G1′ and A3′ still form Watson–Crick-like pairing with C6 and T4 of the complementary strand, G2′ does not form regular Watson-Crick pairing with C5. The nucleobases of G2′ and C5 only forms one H-bond, which is between their O6 and N3 atoms, respectively. Phe173 is located in the middle of the α3 helix and its side chain stacks against the A3′-T4 pair of DNA3 (Figure 4B). Unlike a regular DNA duplex, the nucleobases of both A3 and T4 are slightly tilted toward the T4′-A3 base pair. Instead of stacking, the side chain of Phe173 directly points toward G2′ and C5, which leads to almost 90° rotation for both nucleotides. G1′ and C6 pair with each other; however, compared to a regular Watson–Crick G–C pair, the average H-bond distance (3.15 Å) between G1′ and C6 is ∼0.3 Å longer. The weaker G1′–C6 pair and the irregular G2′–C5 pair all indicate that DNA3 is unwound to some extent.

To investigate the functional role of Phe173, we constructed one F173A mutant of *Hs*MGME1 and carried out an *in vitro* cleavage assay. As depicted in Figure 4C, replacing Phe173 with Ala173 clearly weakened the protein's degradation activity toward all tested duplex-containing DNAs (Supplementary Table S3), including double-stranded DNA4 (dsDNA4), 3′-overhang DNA4, and 5′-overhang DNA4. These observations indicated that Phe173 can facilitate the unwinding and degradation of duplex-containing DNAs by *Hs*MGME1. Interestingly, mutation of Phe173 has no strong impact on single-stranded DNA (such as ssDNA1) cleavage by *Hs*MGME1. Like the WT protein, F173A mutant can efficiently cleave ssDNA1; at low protein concentration (0.05 μmol), the ssDNA1 cleavage activity of F173A is even slightly higher than that of WT *Hs*MGME1.

Interestingly, though it is important for duplex-containing DNA degradation, Phe173 adopts a similar conformation in the *Hs*MGME1-DNA3 and *Hs*MGME1-ssDNA2 complexes (Supplementary Figure S3B). Like Phe173, Trp152 also adopts a similar conformation in the two structures (Supplementary Figure S3C). Trp152 is located at the single strand and duplex junction region of DNA3; its side chain is sandwiched by the sugar pucker of C6 on one side and by the side chains of Met156 and Phe165 on the other side. The strong hydrophobic interactions (as short as 3.55 Å) between Trp152 and the two hydrophobic residues fixed the conformation against the ribose. Compared to A8 of the *Hs*MGME1-ssDNA2 complex, the nucleobase of C6 is slightly tilted toward Trp152 in *Hs*MGME1-DNA3. Due to its close contact with Trp152, C6 could not undergo further movement, which may lead to the weak C6-G1′ pairing. Like WT and F173A mutant of *Hs*MGME1, the *in vitro* cleavage assays (Figure 4C) showed that the W152A mutant of *Hs*MGME1 can efficiently cleave ssDNA1; however, compared to Phe173, replacing Trp152 with an Ala residue caused more dramatic reduction on the protein's cleavage activities toward duplex-containing DNAs (especially dsDNA4 and 5′-overhang DNA4), suggesting that Trp152 is also important for the function of *Hs*MGME1.

### Functional characterization of the N- and C- terminal regions of *Hs*MGME1

**Figure 5.**
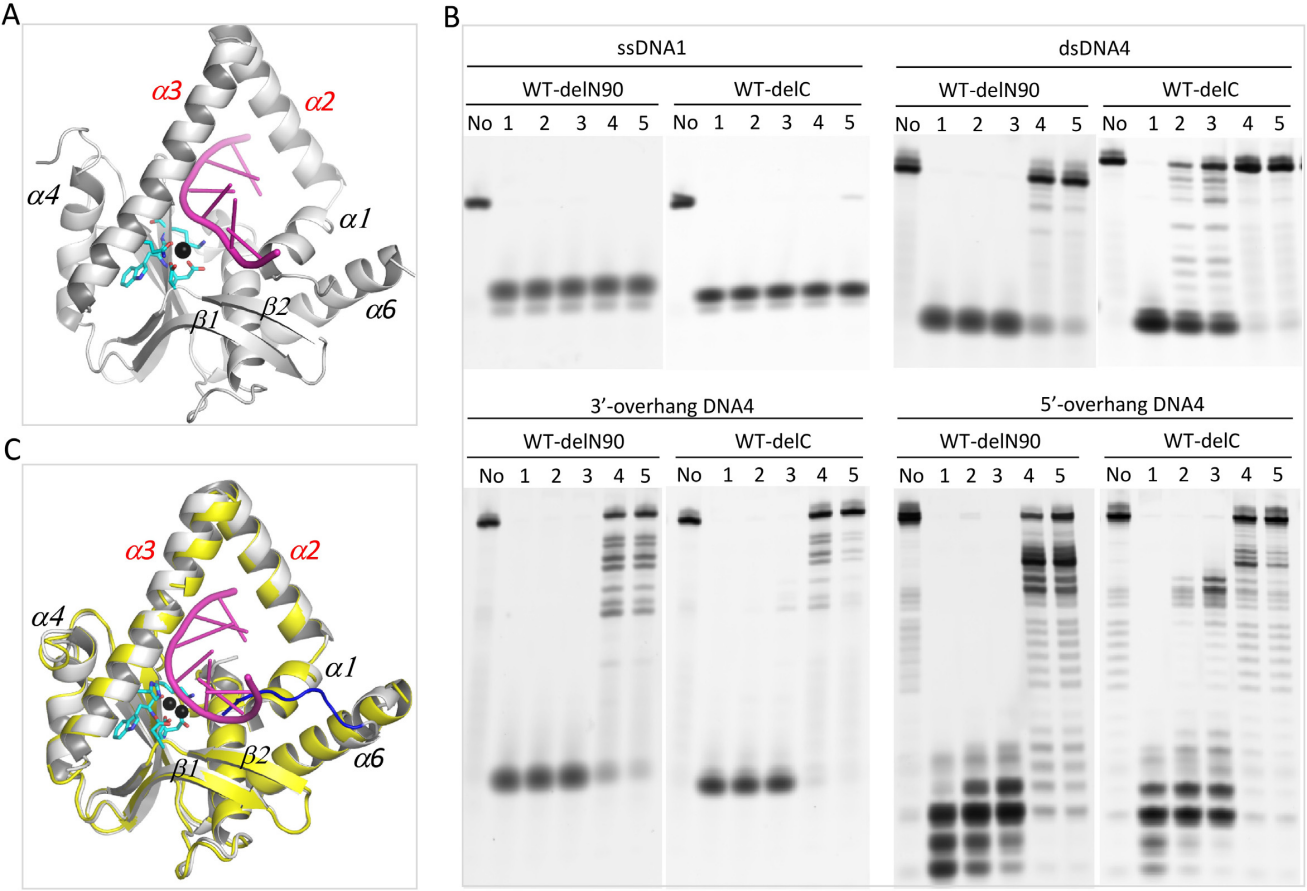
Characterization of the N- and C- terminus of *Hs*MGME1. (**A**) Overall structure of the H180Q-ssDNA2-Ca^2+^ complex. H180Q and Ca^2+^ are shown as cartoon and black sphere, respectively. DNA is shown as cartoon in magenta. (**B**) *in vitro* DNA cleavage assay using N- or C-terminus truncated *Hs*MGME1. (**C**) Superposition of the *Hs*MGME1-Mn^2+^ and *Hs*MGME1-ssDNA2 complexes, showing the conformational change of the C-terminal region. DNA and protein are colored in magenta and yellow in the *Hs*MGME1–ssDNA2 complex. In the *Hs*MGME1–Mn^2+^ complex, Mn^2+^ ions are shown as black spheres, *Hs*MGME1 is shown as cartoon in white, but the C-terminal region is colored in blue. DNA substrate concentrations are 0.8 μM. Truncated *Hs*MGME1 concentrations are 0.8, 0.4, 0.2, 0.1 and 0.05 μM in lanes 1–5, respectively. No protein is present in the lane labelled with No.

None of the crystals contain the N-terminal residues. Around 90 and 130 residues located at the N-terminus of *Hs*MGME1 were disordered in the complex structures of *Hs*MGME1-DNA3 (Figure 4A) and H180Q-ssDNA2-Ca^2+^ (Figure 5A), representing the structures with longest and shortest visible sequences, respectively. Based on these two complex structures, we constructed two truncated *Hs*MGME1 proteins, WT-delN90 and WT-delN130, in which the N-terminal 90 and 130 residues, respectively, were deleted. As revealed by the *in vitro* cleavage assays (Figure 5B), deletion of the 90 N-terminal residues had no impact on ssDNA1 or 3′-flap DNA4 cleavage activities of *Hs*MGME1. Interestingly, compared to WT *Hs*MGME1, WT-delN90 showed slightly higher cleavage activities toward dsDNA4 and 5′-flap DNA4 (Figure 5B).

Unlike WT-delN90 and WT *Hs*MGME1, WT-delN130 is very unstable. As observed in the *Hs*MGME1–ssDNA2 and *Hs*MGME1–DNA3 complex structures, the 90–130 region is very flexible and can switch between two neighboring *Hs*MGME1 molecules. There are four *Hs*MGME1 molecules in the asymmetric unit of *Hs*MGME1-DNA3 complex; arrangement of the four *Hs*MGME1 molecules (Figure 4A) is somewhat similar to the tetrameric structure of *Ec*RecE (42), a nuclease that shares certain sequence similarity with *Hs*MGME1 (Figure 1A) and is responsible for dsDNA free end cleavage during double-stranded DNA break repair process. However, as revealed by our *in vitro* crosslinking assay using WT *Hs*MGME1 (Supplementary Figure S4), no clear bands corresponding to either *Hs*MGME1 dimer or tetramer were generated at lower concentrations (0–250 μM) of DSS (disuccinimidyl suberate). At higher concentrations (500–1000 μM) of DSS, some *Hs*MGME1 dimer and tetramer bands were observed, but the total percentage was very low. These observations suggest that *Hs*MGME1 mainly functions as a monomer and the 90–130 region plays an important role in its stabilization.

In the *Hs*MGME1–Mn^2+^ structure, the C-terminal residues 337–344 of *Hs*MGME1 are well ordered and fold directly back into the active center. Structural superposition (Figure 5C) showed that the C-terminal residues are partially overlapped with the DNA substrate; to avoid their potential conflict with DNA, the C-terminal region is shifted away from the active site and completely disordered in *Hs*MGME1–ssDNA2 and all other *Hs*MGME1–DNA complex structures. In *Hs*MGME1–Mn^2+^, the most C-terminal residue Glu344 is involved in A-site Mn^2+^ coordination (Figure 1D); however, our *in vitro* cleavage assay (Figure 5B) showed that deletion of the C-terminal region (for WT-delC) has no impact on ssDNA1 or 3′-flap DNA4 cleavage, suggesting that Glu344-Mn^2+^ coordination is not critical for the protein's cleavage activity. Compared to the full-length *Hs*MGME1, WT-delC has relatively weaker dsDNA4 or 5′-flap DNA4 cleavage activity, which is in contrast to WT-delN90. The detailed mechanism underlying the inhibitory or enhancing effects of the N- or C-terminal region of *Hs*MGME1 requires further investigation.

## DISCUSSION

*Hs*MGME1 is a recently discovered mitochondrial nuclease. Unlike many other mitochondrial nucleases, *Hs*MGME1 has very broad substrate tolerance with a preference for ssDNA. The detailed ssDNA binding mode of *Hs*MGME1 was revealed by our *Hs*MGME1-DNA2 (Figure 2A) and H180Q-ssDNA2-Ca^2+^ (Figure 5A) complexes. As indicated by both structures, *Hs*MGME1 is an endonuclease that cleaves ssDNA in the middle. Both our *in vitro* assay (Supplementary Figure S4) and a previous study (1) showed that *Hs*MGME1 can cleave 5′ P^32^-labelled ssDNA and generate ladder-like products, indicating that *Hs*MGME1 is also an exonuclease.

*Hs*MGME1 belongs to the PD-(D/E)XK superfamily; in addition to MGME1 orthologues from other species, it also shares sequence similarity with RecE-type and RecB-type nucleases (Figure 1A). Dali search results showed that the structure of MGME1 is most similar to that of *Ec*RecE (PDB_ID: 3H4R) (42), followed by Cas4 nuclease SSO0001 from *Sulfolobus solfataricus* (PDB_ID: 4IC1) (43) and an uncharacterized exonuclease from *Eubacterium rectale* (PDB_ID: 3L0A). MGME1 also shares structural similarity with the structure of phage-related exonuclease (PDB_ID: 3K93) and the structure of K131A mutant of phage lambda exonuclease (PDB_ID: 3SM4) (44), which ranked fifth and sixth on the Dali search result list, respectively. Though the other four structures are all nuclease-alone structures, DNA substrate was present in the phage lambda exonuclease structure. Interestingly, conformations of the DNA and catalytic residues and coordination of the cations (Supplementary Figure S5A) are very similar in the phage lambda exonuclease structure and our substrate DNA binding model of *Hs*MGME1, which further supports the catalytic mechanism we proposed.

Besides ssDNA, MGME1 can also cleave dsDNA and DNA with 5′-flap or 3′-flap. The detailed basis for 3′-overhang DNA binding by *Hs*MGME1 was revealed by the *Hs*MGME1-DNA3 structure (Figure 4A). As depicted in Figure 4B, *Hs*MGME1 will break the O-P bond between the third and fourth nucleotides from the 3′-end of the duplex. Our *in vitro* cleavage assays (Figure 4C) showed that 5′ FAM-labeled dsDNA4 can be cleaved by MGME1 (or MGME1 mutants) from the 3′-end and generate ladder-like products, suggesting that DNA duplex can bind to *Hs*MGME1 in a manner similar to the duplex region of DNA3. MGME1 can also bind 5′-overhang DNA in a similar way with the 5′-overhang simply flipped out. In addition to the catalytic mechanism, structural comparison also revealed that the region corresponding to the *Hs*MGME1 helical arch is very flexible. This region is completely disordered in the *Ec*RecE structure; unlike MGME1, this region is significantly tilted in the phage lambda exonuclease structure, forming one cleft suitable for DNA duplex or 5′-overhang DNA binding (Supplementary Figure S5B). Similar to dsDNA4, ladder-like products were also observed in the presence of MGME1 and 5′-overhang DNA4 (which was FAM-labelled at the 3′-end). These observations suggested that *Hs*MGME1 can undergo large conformational changes, bind, and cleave 5′-overhang DNA from the 5′-end similar to the phage lambda exonuclease.

**Figure 6.**
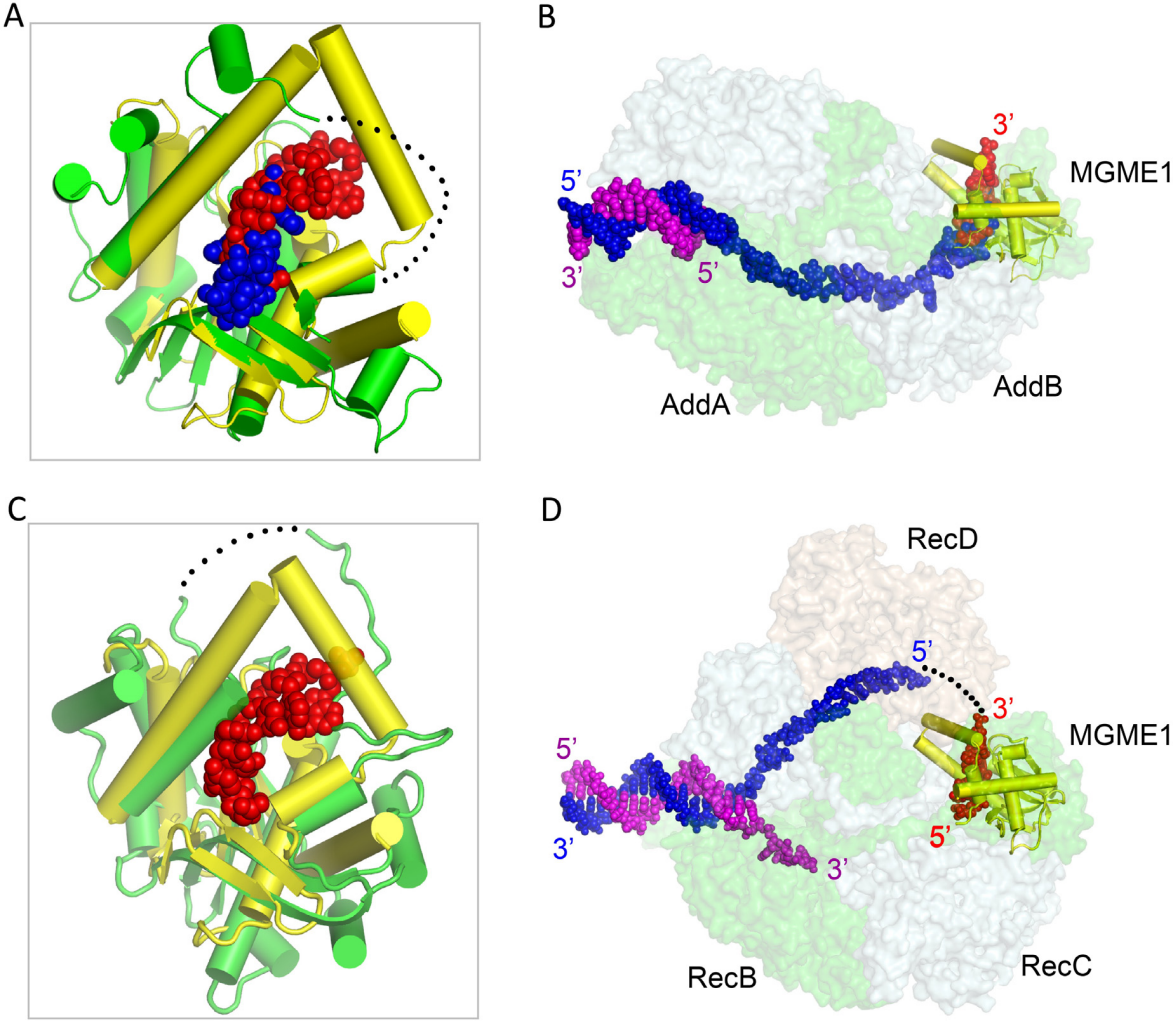
Structural comparison of *Hs*MGME1 and RecB-type nucleases. (**A**) Superposition of *Hs*MGME1-ssDNA2 and the homologous domain from AddA within the AddAB-DNA complex (PDB_ID: 4CEJ). (**B**) Fitting of *Hs*MGME1-ssDNA2 onto the AddAB-DNA complex. (**C**) Superposition of *Hs*MGME1-ssDNA2 and the homologous domain from RecB within the RecBCD-DNA complex (PDB_ID: 1W36). (**D**) Fitting of *Hs*MGME1-ssDNA2 onto the RecBCD-DNA complex. *Hs*MGME1 and ssDNA2 of the *Hs*MGME1-ssDNA2 complex are shown as yellow cartoon and red spheres in all panels. The nuclease domains of AddA and RecB are shown as green cartoons in panels (A) and (C). In panels (B) and (D), RecBCD and AddAB protein complexes are shown as surface, but their substrate DNAs are shown as spheres in blue and magenta. Black dots indicated the residues disordered around the helical arch regions of RecB and AddA, and the possible pathway of substrate DNA 5′-end of RecBCD complex.

Unlike *Ec*RecE, Cas4 nuclease SSO0001, the uncharacterized *E. rectale* exonuclease, phage-related exonuclease, and phage lambda exonuclease, which all function as oligomers, *Hs*MGME1 functions as a monomer. This unique feature sets *Hs*MGME1 apart from other nucleases and contributes to the broad substrate tolerance of *Hs*MGME1. Perhaps due to its ability to cleave various substrates, *Hs*MGME1 participates in different biological processes of mtDNA, including replication and double-strand degradation. *Hs*MGME1 shares sequence similarity with RecB-type nucleases, including AddA and RecB (Figure 1A). AddA ranked fourth on the Dali search result list; it is the catalytic component of DNA double-strand break repair complex AddAB that trims DNA in the 3′→5′ direction (27). Structural comparison (Figure 6A and B) could clarify the substrate binding and catalytic mechanism of AddAB. As revealed by the recent *Bs*AddAB-DNA complex structure (PDB_ID: 4CEJ), the 3′-end of the DNA is located near the active site of AddA. However, conformations of the local DNAs are significantly different in the AddAB-DNA complex and our H180Q-ssDNA2–Ca^2+^ complex (Supplementary Figure S6A) or *Hs*MGME1–ssDNA2 complex, owing to disordering of the helical arch and mutation of catalytic residue Asp1172. In addition to catalysis, these observations suggest that proper coordination of cations is also important for catalytic complex assembly.

RecB ranked seventh on the Dali search result list; it is the catalytic component of another DNA double-strand break repair complex, RecBCD. Upon binding of the chi sequence, RecB will trim the overhang regions of substrate DNAs in the 5′→3′ direction. Several structures of RecBCD in complex with DNA have been reported previously (45), but no DNA was captured at or near the active site of RecB (Figure 6C). In one RecBCD–DNA complex structure (PDB_ID: 1W36), one Ca^2+^ coordinated with the catalytic site residues of RecB; coordination of Ca^2+^ is very similar to Mn^2+^ at the A-site of the *Hs*MGME1–Mn^2+^ structure (Supplementary Figure S6B) and Ca^2+^ in the H180Q–ssDNA2–Ca^2+^ complex, suggesting that RecBCD and *Hs*MGME1 may follow a similar mechanism in cation binding and catalysis. Perhaps due to the absence of interacting DNA, RecB does not form a helical arch in any RecBCD structures; however, structural analysis (Figure 6D) suggests that RecB could undergo large conformational changes to create a helical arch similar to the one in the *Hs*MGME1–DNA complex. The helical arch may play an important role in substrate binding and catalytic process.

*Hs*MGME1 has one unique structural pin (Figure 4B and C) that can facilitate DNA unwinding and enhance the cleavage efficiency of duplex-containing DNA by *Hs*MGME1. Instead of the homologous protein RecB, one pin motif was identified in RecC previously; though the detailed conformations of the pins are not identical in *Hs*MGME1 and RecC, they may play similar roles in DNA unwinding. Besides the structural pin, all the components of RecBCD and AddAB complexes also contain helicase domains; precise incorporation among these helicases are essential for substrate DNA unwinding and repair of the breaks. *Hs*MGME1 does not contain a helicase domain, but *Hs*MGME1, PolgA, and TWINKLE (a mitochondrial DNA helicase) can work together in the replication and double-strand degradation process of mtDNA (21,22). In the future, it is worth investigating whether TWINKLE can mimic the nuclease domains of RecBCD (or AddAB) and form stable complexes with *Hs*MGME1 and/or PolgA to ensure the rapid degradation of damaged mtDNAs.

7S DNA is the product of prematurely terminated replication of mtDNA, initiated at the NCR region; during the replication process, the NCR region and 7S DNA form a D-loop structure. Depending on the species, the size and sequence of the 5′-end of 7S DNA could been very different (18) and can be very rich in TMP or AMP (close to 50%). Both our results and previous *in vitro* cleavage assays all confirmed that *Hs*MGME1 can cleave ssDNAs in either the 5′→3′ or 3′→5′ direction. However, *in vivo* studies showed that loss of *Hs*MGME1 mainly led to the accumulation of 7S DNA with elongated 5′-ends in mitochondria. The detailed structural basis for the cleavage site preference and *in vivo* 5′-end preference of 7S DNA degradation of *Hs*MGME1 are still not clear. The N- and C-terminal region of *Hs*MGME1 are very flexible and have certain enhancing or inhibitory impacts on *in vitro* DNA degradation by *Hs*MGME1 (Figure 5B). As reveled by structural comparison, the β3-α5 loop could also undergo large conformational changes (Supplementary Figure S1C). It is worth investigating whether these flexible regions of *Hs*MGME1 is involved in PolgA and TWINKLE interactions and contributes to the *in vivo* 7S DNA degradation directionality of *Hs*MGME1.

In summary, we solved four high resolution structures of *Hs*MGME1 in this study. Our structures revealed the detailed basis for various DNA binding by *Hs*MGME1. In combination with *in vitro* cleavage assays and structural analysis, our structures also reveal the possible catalytic mechanism for *Hs*MGME1 and homologous proteins, including AddA and RecB of the DNA double-strand break repair complex AddAB and RecBCD. Unlike many other mitochondrial nucleases present in both mitochondria and other cellular organelles, MGME1 is only found in mitochondria. Though how *Hs*MGME1 mutations lead to the associated mitochondrial diseases needs to be further investigated, our *Hs*MGME1 structures provided one potential target in regulating DNA replication and double-strand degradation processes specific to mitochondria.

## DATA AVAILABILITY

Structure factors and coordinates have been deposited in the Protein Data Bank under accession codes 5ZYW, 5ZYU, 5ZYV and 5ZYT for the *Hs*MGME1–Mn^2+^, *Hs*MGME1–ssDNA2, H180Q–ssDNA2–Ca^2+^ and *Hs*MGME1–DNA3 complexes, respectively.

## Supplementary Material

Supplementary Data
