# Supplementary material for: Structural insights into DNA degradation by human mitochondrial nuclease MGME1
Source: Nucleic Acids Res. 2018 Sep 21;46(20):11075–88. doi: 10.1093/nar/gky855 (PMC6237815; doi:10.1093/nar/gky855)
Supplement: Supplementary Data [file gky855_supplemental_files.pdf]

Supplementary Data

For

Structural insights into DNA degradation by human mitochondrial nuclease MGME1

Chun Yang[1], Ruiqi Wu[1], Hehua Liu[1,2], Yiqing Chen[1], Yanqing Gao[1], Xi Chen[1], Yangyang Li[1], Jinbiao Ma[2], Jixi Li[1,3*], Jianhua Gan[1,*]

[1]State Key Laboratory of Genetic Engineering, Collaborative Innovation Center of Genetics and Development, Department of Physiology and Biophysics, School of Life Sciences, Fudan University, Shanghai, 200433, China

[2]State Key Laboratory of Genetic Engineering, Collaborative Innovation Center of Genetics and Development, Department of Biochemistry, School of Life Sciences, Fudan University, Shanghai, 200433, China

[3]Department of Neurology, Huashan Hospital, Fudan University, Shanghai, 200040, China

***Correspondence to**: ganjhh@fudan.edu.cn or lijixi@fudan.edu.cn

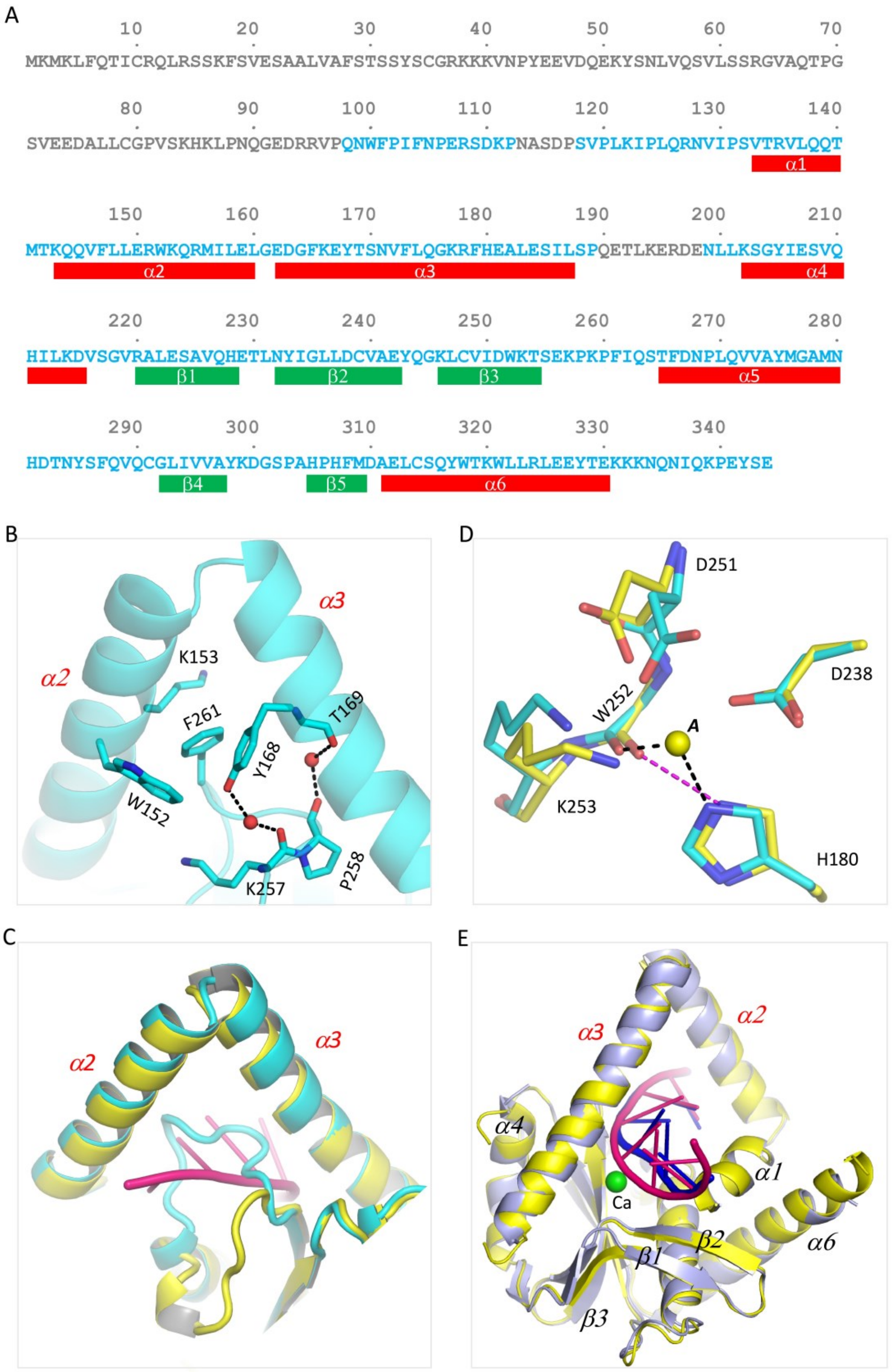

Supplementary Figure S1. Conformational changes of *Hs*MGME1. (**A**) Sequence and secondary structure of *Hs*MGME1. Residues disordered in *Hs*MGME1-Mn^{2+} complex are colored in gray. (**B**) Detailed conformations of the helical arch and β3-α5

connecting loop observed in the *Hs*MGME1-Mn^{2+} complex. (**C**) Superposition of *Hs*MGME1-Mn^{2+} and *Hs*MGME1-ssDNA2 complexes showing the conformational change of the β3-α5 connecting loops. (**D**) Comparison of the catalytic site residues of *Hs*MGME1-Mn^{2+} and *Hs*MGME1-ssDNA2. (**E**) Superposition showing the local conformational difference of the DNAs, which are shown as magenta and blue cartoons in *Hs*MGME1-ssDNA2 and H180Q-ssDNA2-Ca^{2+} complexes, respectively. Water molecules, Mn^{2+} and Ca^{2+} are shown as red, yellow, and green spheres, respectively.

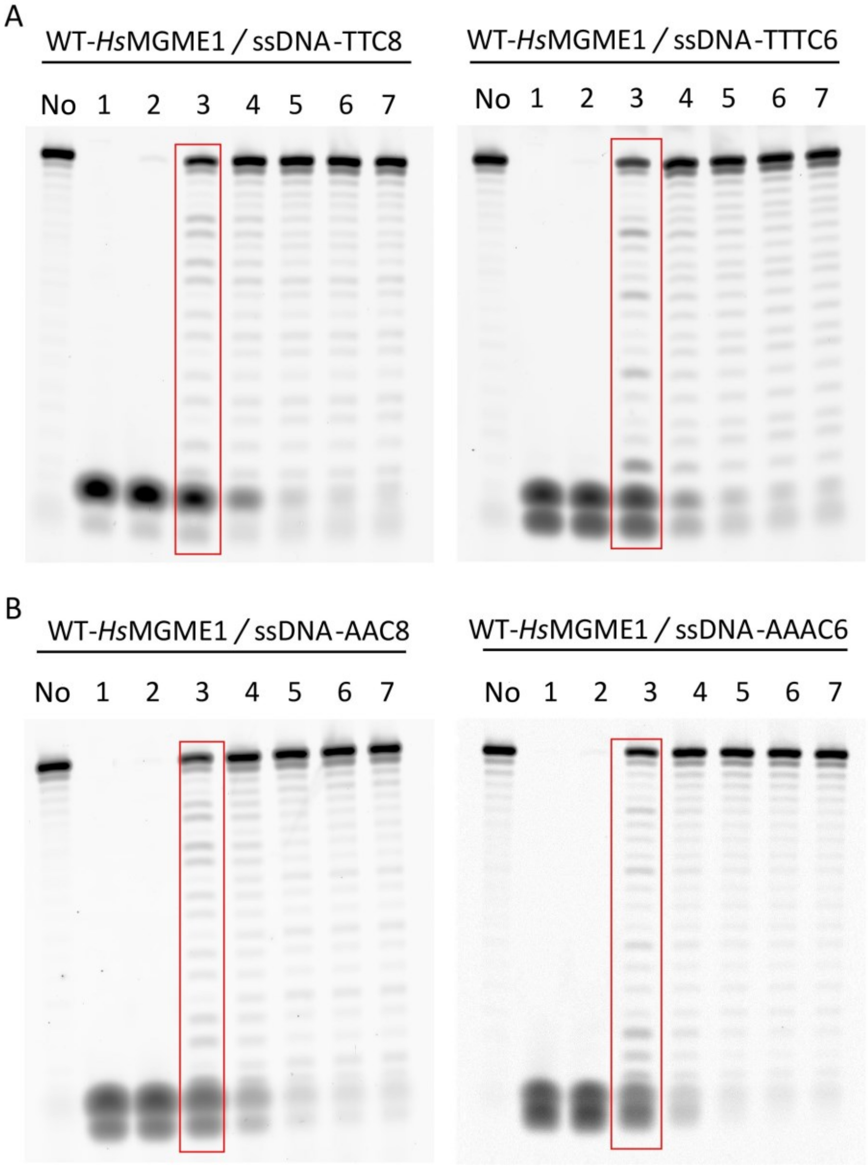

Supplementary Figure S2. The weak cleavage site preference of *Hs*MGME1. (**A**) *in vitro* ssDNA-TTC8 and ssDNA-TTTC6 cleavage by WT *Hs*MGME1. (**B**) *in vitro* ssDNA-AAC8 and ssDNA-AAAC6 cleavage by WT *Hs*MGME1. DNA concentrations are 0.8 μM. Protein concentrations are 0.8 μM, 0.4 μM, 0.2 μM, 0.1 μM, 0.05 μM, 0.025 μM, and 0.0125μM in lanes 1-7, respectively. *Hs*MGME1 is absent in the lane labelled with No.

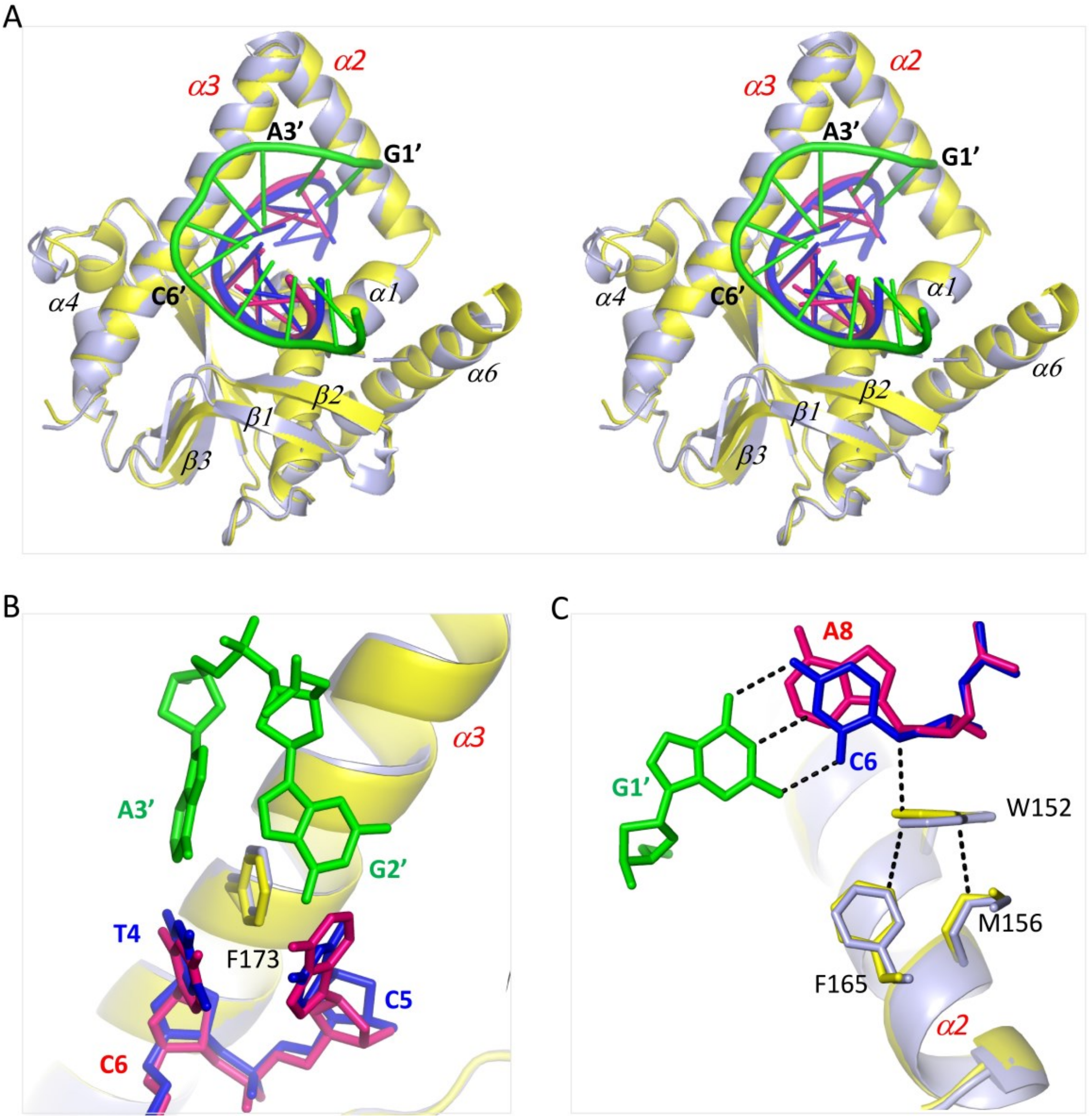

Supplementary Figure S3. Comparison of single-stranded and duplex DNA bound *Hs*MGME1. (**A**) Stereoview showing the comparison of *Hs*MGME1-ssDNA2 and *Hs*MGME1-DNA3 complexes. (**B**) and (**C**) Conformational comparison of the pin residues and the surrounding nucleotides. For *Hs*MGME1-DNA2, the protein and DNA are colored in yellow and magenta. For *Hs*MGME1-DNA3, the protein and the two DNA strands are colored in light-blue, green, and blue, respectively. The sequences of ssDNA2 (5’-AACAACAACAAC-3’) and DNA3 (5’-GGATCCTTCTTCTTCTTC -3’) have no coincidence.

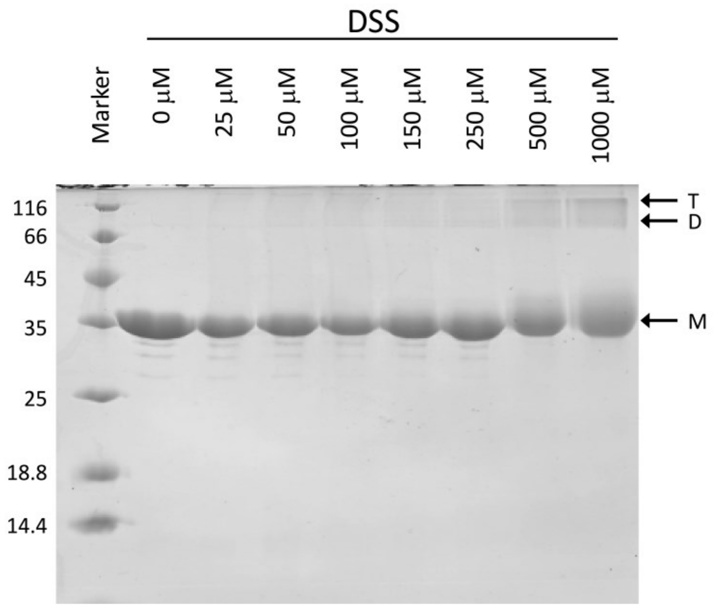

Supplementary Figure S4. DSS crosslinking analysis of *Hs*MGME1. Protein and DNA3 concentrations are 50 μM and 50 μM, respectively. DSS concentrations are indicated on the gel. The bands corresponding to the monomer, dimer, and tetramer of *Hs*MGME1 are indicated by arrows and labelled as M, D, and T, respectively.

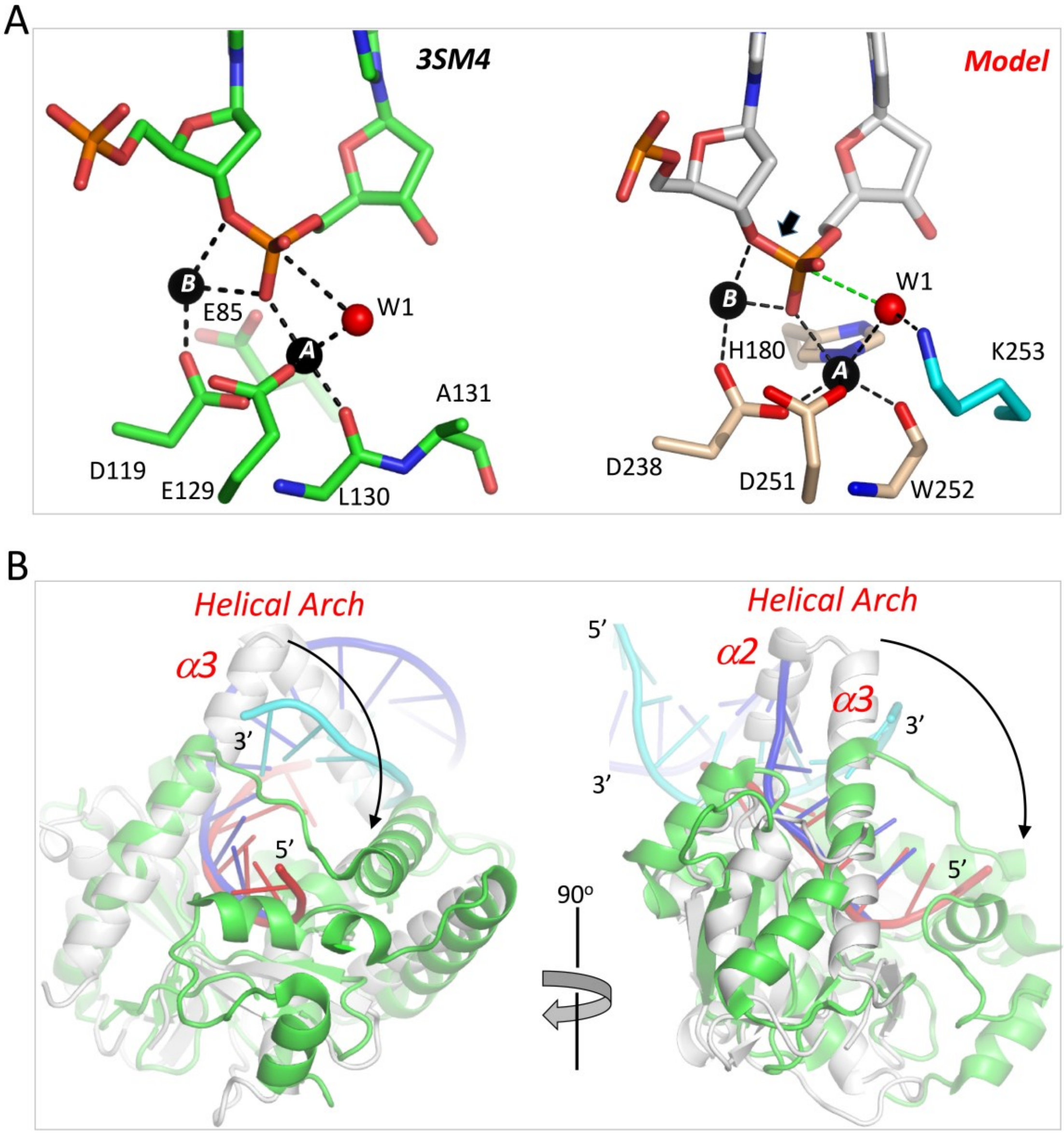

Supplementary Figure S5. Structural comparison between *Hs*MGME1 and phage lambda nuclease. (**A**) Comparison of the active site structure of the K131A mutant of phage lambda nuclease (PDB_ID: 3SM4) and the catalytic MGME1 model we proposed. Cations and the catalytic water molecule are shown as spheres in black and red, respectively. (**B**) Structural superposition showing the flexibility of the regions corresponding to the helical arch of *Hs*MGME1. *Hs*MGME1 and the phage lambda nuclease are colored in white and green, respectively. DNA is colored in red in *Hs*MGME1-DNA2 structure, whereas it is colored in blue and cyan in the phage lambda nuclease structure.

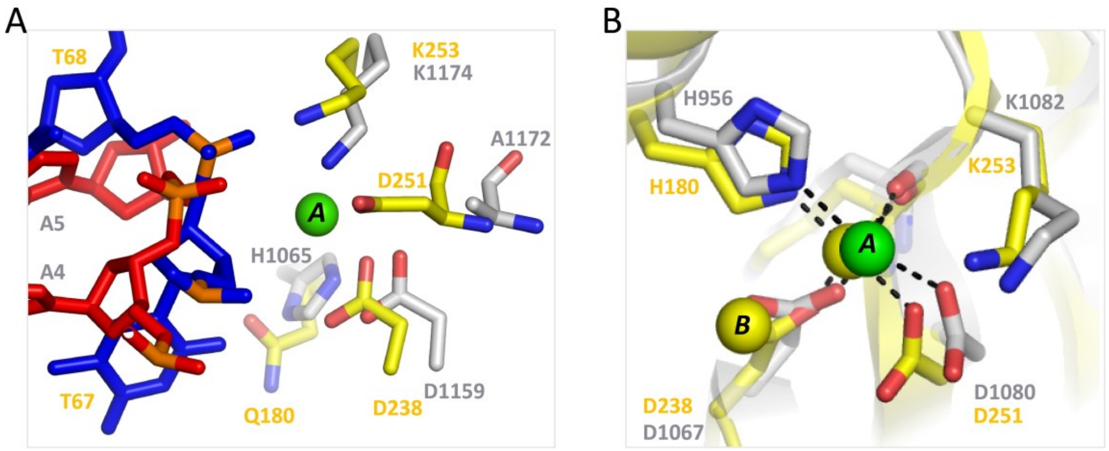

Supplementary Figure S6. Structural comparison between *Hs*MGME1 and RecB-type nucleases. (**A**) Structural superposition showing the different conformations of DNAs near the cleavage sites of H180Q-ssDNA2-Ca^{2+} and AddAB-DNA complexes. Ca^{2+} is shown as green sphere in H180Q-ssDNA2-Ca^{2+}. The DNA C-atoms are colored in red and blue in the H180Q-ssDNA2-Ca^{2+} and the AddAB-DNA complexes, respectively. *Hs*MGME1 H180Q mutant and RecB are shown as sticks with their C-atoms colored in yellow and white, respectively. (**B**) Structural superposition showing the similar coordination of cations bound in the active sites of the *Hs*MGME1-Mn^{2+} and the RecBCD-DNA complexes. Mn^{2+} of *Hs*MGME1-Mn^{2+} and Ca^{2+} of RecBCD-DNA are shown as spheres in yellow and green, respectively. *Hs*MGME1 and D1172A mutant of AddA are all shown as sticks with their C-atoms colored in yellow and white, respectively.

Supplementary Table S1. Sequences of the primers used for WT and mutant *Hs*MGME1 construction

Name	Sequence [a]
Full-length MGME1-F	CGC*GGATCC*GGTGGTGGTATGAAGATGAAGTTATTTCAGACCATTTGCAGGCAG
WT-F	CGC*GGATCC*GAATCAGCTGCCCTTGTGGC
delN90-F	CGC*GGATCC*GGTGAGGACAGACGAGTGCCAC
delN130-F	CGC*GGATCC*GGTGGTGGTATACCAAGTGTGACCCGAGTCCTTCAGC
WT-R	AAA*CTCGAG*CTATTCTGAATATTCTGGTTTCTGAATATTC
WT-delC-R	AAA*CTCGAG*CTATTTCTTTTCCGTATATTCTTCTAGTC
T134A-F	GTGATACCAAGTGTGGCCCGAGTCCTTCAGCAG
T134A-R	CTGCTGAAGGACTCGGGCCACACTTGGTATCAC
Q145A-F	CCATGACAAAACAAGCTGTTTTCTTGTTGGAGAGG
Q145A-R	CCTCTCCAACAAGAAAACAGCTTGTTTTGTCATGG
W152A-F	GTTTTCTTGTTGGAGAGGGCTAAACAGCGGATG
W152A-R	CATCCGCTGTTTAGCCCTCTCCAACAAGAAAAC
F173A-F	GAATACACTTCAAACGTCGCTTTACAAGGGAAACGGTTC
F173A-R	GAACCGTTTCCCTTGTAAAGCGACGTTTGAAGTGTATTC
H180Q-F	GGGAAACGGTTCCAAGAAGCCTTGGAAAG
H180Q-R	CTTTCCAAGGCTTCTTGGAACCGTTTCCC
E184Q-F	CCACGAAGCCTTGCAAAGCATACTTTCACCCC
E184Q-R	GGGGTGAAAGTATGCTTTGCAAGGCTTCGTGG
E223Q-F	GGAGTGCGAGCTCTTCAAAGTGCTGTTCAACATG
E223Q-R	CATGTTGAACAGCACTTTGAAGAGCTCGCACTCC
D238N-F	CTATATAGGTCTGCTGAACTGTGTGGCTGAGTATC
D238N-R	GATACTCAGCCACACAGTTCAGCAGACCTATATAG
D251N-F	GCAAGCTCTGTGTGATTAATTGGAAGACATCAGAG
D251N-R	CTCTGATGTCTTCCAATTAATCACACAGAGCTTGC
T254A-F	GTGTGATTGATTGGAAGGCTTCAGAGAAACCAAAG
T254A-R	CTTTGGTTTCTCTGAAGCCTTCCAATCAATCACAC
F266A-F	GCCTTTTATTCAAAGTACAGCTGACAACCCACTGC
F266A-R	GCAGTGGGTTGTCAGCTGTACTTTGAATAAAAGGC
Q271A-F	GACAACCCACTGGCTGTTGTGGCATACATGGG
Q271A-R	CCCATGTATGCCACAACAGCCAGTGGGTTG TC
Y275A-F	CTGCAAGTTGTGGCAGCTATGGGTGCCATGAAC
Y275A-R	GTTCATGGCACCCATAGCTGCCACAACTTGCAG

[a]GGATCC and CTCGAG highlighted with underline are BamHI and XhoI recognition sequence.

Supplementary Table S2. Data collection and refinement statistics [a]

Structure (PDB ID)	MGME1-Mn^{2+} 5ZYW	MGME1-DNA2 5ZYU	H180Q-DNA2-Ca^{2+} 5ZYV	MGME1-DNA3 5ZYT	*Se*-MGME1-DNA3
**Data collection**[a]					
Space group	$P4_32_12$	$P3_2$	$P2_12_12$	C2	C2
Cell parameter:					
a (Å)	68.0	80.7	81.3	146.5	146.3
b (Å)	68.0	80.7	106.2	77.5	78.2
c (Å)	114.8	79.8	33.4	164.7	165.7
β (°)	90	90.0	90.0	112.4	112.4
Wavelength (Å)	0.9793	0.9793	0.9793	0.9793	0.9793
Resolution (Å)	30.0-2.20	30.0-1.75	30.0-2.70	30.0-2.70	30.0-3.0
Last shell (Å)	2.28-2.20	1.81-1.75	2.80-2.70	2.80-2.70	3.05-3.0
Completeness (%)	100.0(100.0)	100.0(100.0)	97.6(86.8)	94.6(90.6)	99.9(100.0)
Redundancy	21.6(14.8)	9.6(8.0)	7.5(3.4)	5.6(5.1)	6.6(6.5)
I/σ(I)	31.0(2.5)	16.4(2.3)	17.3(2.7)	15.4(2.2)	16.0(2.1)
Rmerge (%)	10.5(48.0)	9.4(47.7)	9.5(35.6)	9.4(46.4)	12.7(69.5)
**Refinement**					
Resolution (Å)	29.9-2.20	28.4-1.75	28.3-2.72	30.0-2.70	
R_{work} (%) / R_{free} (%)	22.6/25.2	17.4/20.0	21.2/25.4	25.0/29.6	
No. of atoms					
Protein	1854	3541	1555	7164	
DNA		290	126	872	
Cations	3		2		
water	94	532	13		
R.m.s. deviations					
Bond length (Å)	0.007	0.011	0.004	0.010	
Bond angle (°)	1.113	1.036	0.613	1.250	
Ramachandran plot (%)					
Most favored	98.7	98.6	95.3	96.0	
Additional allowed	1.3	1.4	4.7	4.0	

[a]: Values in parentheses are for the last resolution shell.

Supplementary Table S3. Sequences of DNAs used in crystallization and *in vitro* cleavage assays

Name	Sequence (5'-3')
**DNAs for crystallization**	
ssDNA2	AACAACAACAAC
DNA3	GGATCCTTCTTCTTCTTC
**DNAs for *in vitro* cleavage assay** [a]	
ssDNA1	TTCTTCTTCTTC
ssDNA2	AACAACAACAAC
dsDNA4	GGATGGGGGATCCCCCATCC
5'-overhang DNA4	TTCTTCTTCTTCGGATGGGGGATCCCCCATCC
3'-overhang DNA4	GGATGGGGGATCCCCCATCCTTCTTCTTCTTC
ssDNA-TTC8	TTCTTCTTCTTCTTCTTCTTCTTC
ssDNA-TTTC6	TTTCTTTCTTTCTTTC
ssDNA-AAC8	AACAACAACAACAACAACAACAAC
ssDNA-AAAC6	AAACAAACAAACAAACAAACAAAC

[a]: Except 5'-overhang DNA4 that was FAM-labelled at the 3'-end, all other DNAs are FAM-labelled at their 5'-ends. The sequences that can form self-complementary duplexes are highlighted with underlines.
